# Hip-spine relationship: clinical evidence and biomechanical issues

**DOI:** 10.1007/s00402-024-05227-3

**Published:** 2024-03-12

**Authors:** Alberto Di Martino, Giuseppe Geraci, Matteo Brunello, Claudio D’Agostino, Giorgio Davico, Cristina Curreli, Francesco Traina, Cesare Faldini

**Affiliations:** 1 Ist Orthopaedic Department, IRCCS-Istituto Ortopedico Rizzoli, Via Giulio Cesare Pupilli, 1, 40136 Bologna, Italy; 2 Department of Biomedical and Neuromotor Science-DIBINEM, University of Bologna, 40136 Bologna, Italy; 3 Medical Technology Lab, IRCCS Istituto Ortopedico Rizzoli, Bologna, Italy; 4 Department of Industrial Engineering, Alma Mater Studiorum - University of Bologna, Bologna, Italy; 5 Ortopedia-Traumatologia e Chirurgia Protesica e dei Reimpianti di Anca e di Ginocchio, IRCCS Istituto Ortopedico Rizzoli, Bologna, Italy

**Keywords:** Biomechanics, Computational modelling, Hip spine, Bone, Total hip arthroplasty

## Abstract

The hip-spine relationship is a critical consideration in total hip arthroplasty (THA) procedures. While THA is generally successful in patient, complications such as instability and dislocation can arise. These issues are significantly influenced by the alignment of implant components and the overall balance of the spine and pelvis, known as spinopelvic balance. Patients with alteration of those parameters, in particular rigid spines, often due to fusion surgery, face a higher risk of THA complications, with an emphasis on complications in instability, impingement and dislocation. For these reasons, over the years, computer modelling and simulation techniques have been developed to support clinicians in the different steps of surgery. The aim of the current review is to present current knowledge on hip-spine relationship to serve as a common platform of discussion among clinicians and engineers. The offered overview aims to update the reader on the main critical aspects of the issue, from both a theoretical and practical perspective, and to be a valuable introductory tool for those approaching this problem for the first time.

## Introduction

**Fig. 1:**
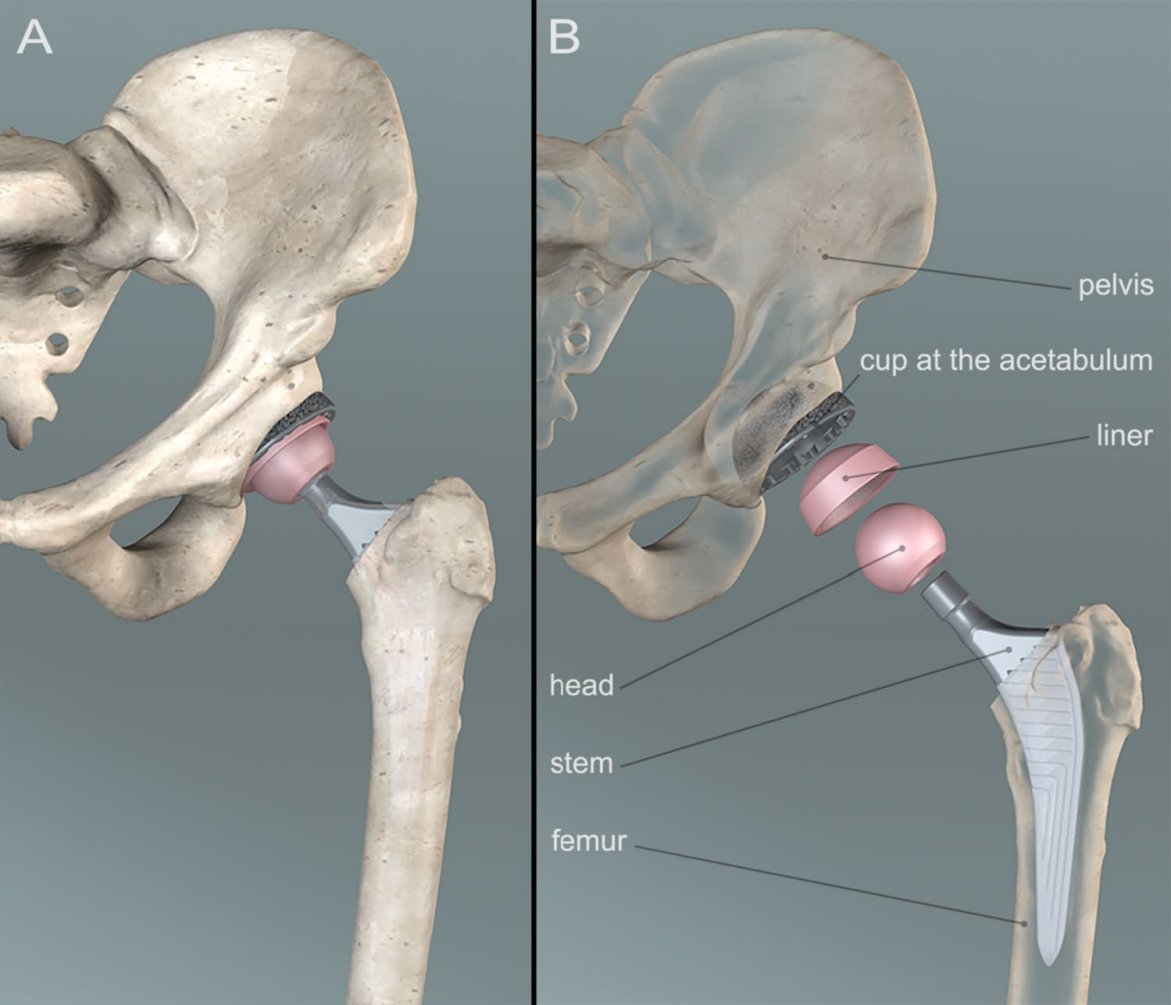
**A** 3D image of a Total Hip Arthroplasty (THA), as the implant appears in a real condition. **B** The prosthesis components are divided in fixed and mobile: the fixed are the cup, that is placed in the pelvis, and the stem that is placed in the femoral canal. The mobile components are the liner that is placed in the cup and the head, that is structured with the stem

Total hip arthroplasty (THA) is performed to treat end-stage osteoarthritis (OA), either primary or secondary, when other surgical or conservative strategies have failed to improve hip function and to control pain, usually when symptoms compromise patient's quality of life [1]. THA consists in the removal of the femoral head and neck and reaming of the acetabular cavity affected by OA, and in the implantation of prosthetic components, namely the femoral stem, the femoral head, and the acetabular cup, thereby constituting a ball and socket type of device (Fig. 1). The design may vary based on clinical indications and healthcare settings, with options including cemented or press-fit components, the latter fitting into the surrounding bone through precise dimensional interferences [2].

THA remains one of the most cost-effective and successful orthopaedic procedures [1] despite the possibility of complications, e.g., instability and dislocation, that can negatively impact on patients’ outcomes and satisfaction [3]. Instability is defined as the temporary and incomplete loss of articulation contact of prosthetic components, with variable but generally benign clinical outcomes, whereas dislocation refers to a clinically dramatic event characterized by the complete loss articular contact between the femoral head and the acetabular cup. Hip instability and dislocation following THA were originally related to obsolete technology, where primitive implants required a small femoral head for longer performance against wear and osteolysis [4]. Furthermore, the wear of older-generation prosthetic components could lead to the loosening of the musculotendinous apparatus and, consequently, a potentially developmental joint laxity that could lead to dislocation [5, 6]. Currently, with advanced prosthetic implants, thanks to the application of tribological concepts in material engineering and prosthetic design, wear is extremely reduced, and dislocation is mostly dependant on the reciprocal relationship among implant components and the overall body balance. The former is called “combined anteversion” and poses limits to cup and femoral anteversion to decrease the risk of impingement and dislocation [7, 8]. Body balance, on the other side, is influenced by the relationship among spine and pelvis, and it has recently been named “spinopelvic balance”: if abnormal, patients show abnormal pelvis anteversion during standing and sitting, and THAs are at risk of impingement because of the mechanical conflict between two prosthetic components, between bone segments, or between a bone segment and a prosthetic component; impingement may cause a levering effect resulting in excessive wear and risk of implant dislocation. Spinopelvic balance is a complex mechanism that is influenced by spinopelvic version and kinematic; indeed, a relevant contribution to human balance is given by mobility on the sagittal plane, where flexion and extension movements are coordinated and shared between the lumbar spine and pelvis, defining a peculiar biomechanical concert [9, 10]. When contribution of the spine to movement is deficient or absent because of stiffness, hip and pelvis must compensate for this deficiency with a greater range of movement to ensure the performance of activities of daily living [11]. Another relevant scenario can be determined by an abnormal positioning of the pelvis on the sagittal plane, potentially being in pathologica and fixed retroversion or anteversion (rare), determining an imbalance in the coordinated movement between the spinopelvic complex and the hip. In either cases, pathologic motion of THA may lead to prosthetic impingement and, potentially, implant dislocation.

In 1978, Lewinnek et al. [12] theorized a “*safe zone*” to place the cup of THA implants to reduce the risk of impingement and dislocations. However, subsequent studies showed that patients with THA within the safe zone still had dislocations [13, 14] because the safe zone itself was meant as a “static value”; this is only partially correct because the modifications in pelvic version on the sagittal plane secondary to spine diseases influences the orientation of the acetabulum [15] from standing to sitting [16, 17]. The hip is mostly influenced by the lumbar vertebrae; therefore, the spine-pelvis-hip relationship can be captured on a lateral radiograph that includes the L1-3 vertebra to the proximal part of the femur [18].

Spinal mobility may be compromised in rigid spines due to degenerative or developmental diseases, or after a spinal fusion surgery has been performed [19, 20]. In the last decade, spine fusion or spine stiffness are more frequently observed in patients candidate to THA. Analyzing the epidemiology of THAs performed every year, approximately 330,000 in the US and approximately 59,000 in Italy, approximately 1% are performed on patients with stiff or fused lumbar spine (LSF) [21]. Several studies reported a higher risk of mechanical complications of THA implants in population operated on for LSF due to partial compromise of spinopelvic kinematics that pushes the compensation mechanisms to the limit [16]. This clinical finding supported studies on the hip-spine relationship to provide hip surgeons resources and tools to understand and adequately manage THA patients affected by diseases at the lumbar spine [22]. In these studies, patients operated on for LSF reported a higher incidence of mechanical failure at long term follow up; the same was not observed in patients undergoing non fusion surgery [23]. Moreover, it was found that failure of THA implants in patients with previous fusion surgery tended to occur in the first two years after THA surgery, confirming the importance of overall implant positioning and patient alignment to determine implant-related complications.

The aim of the current review is to present current knowledge on hip-spine relationship to serve as a common platform of discussion among clinicians and engineers. The offered overview aims to update the reader to with the main critical aspects of the issue, from both a theoretical and practical perspective, and to be a valuable introductory tool for those approaching this problem for the first time.

## Spinopelvic alignment

Under physiological conditions, the spinal column is straight in the frontal plane, while it presents four physiological curves in the sagittal plane: two anterior convexities at the cervical and lumbar level, called lordosis, and two posterior convexities at the dorsal and sacral level, known as kyphosis [24].

There are no absolute standard values for sagittal curves in the adult spine; however, thoracic kyphosis (TK) usually ranges between 20° and 45°, and lumbar lordosis (LL) in most patients ranges between 30° and 60°, with substantial physiological variability across people, or within the same subject depending on age. The lack of defined values of normality derives from the spinal column's primary purpose of maintaining an upright posture with minimal energy demand; moreover, LL is strictly depending on pelvis width, numerically represented by the angle of pelvic incidence, and from pathology at the lumbosacral junction, as in spondylolisthesis [25].

Regardless of the value of the individual curve, in a balanced spine, global compensation is maintained with the center of gravity aligned between the centroid of the body of the last cervical vertebra and the sacrum, the so called Sagittal Vertical Axis (SVA). Sagittal spine curves make it possible to maintain balance and to evenly distribute the load of body weight by transferring it from the spine to the lower limbs, and then to the ground [26]; therefore, the global assessment and the relationships between the various segments of the spine, and between lumbar spine and pelvis, is more important than the quantitative analysis of individual angles for the evaluation of sagittal vertebral balance.

**Fig. 2:**
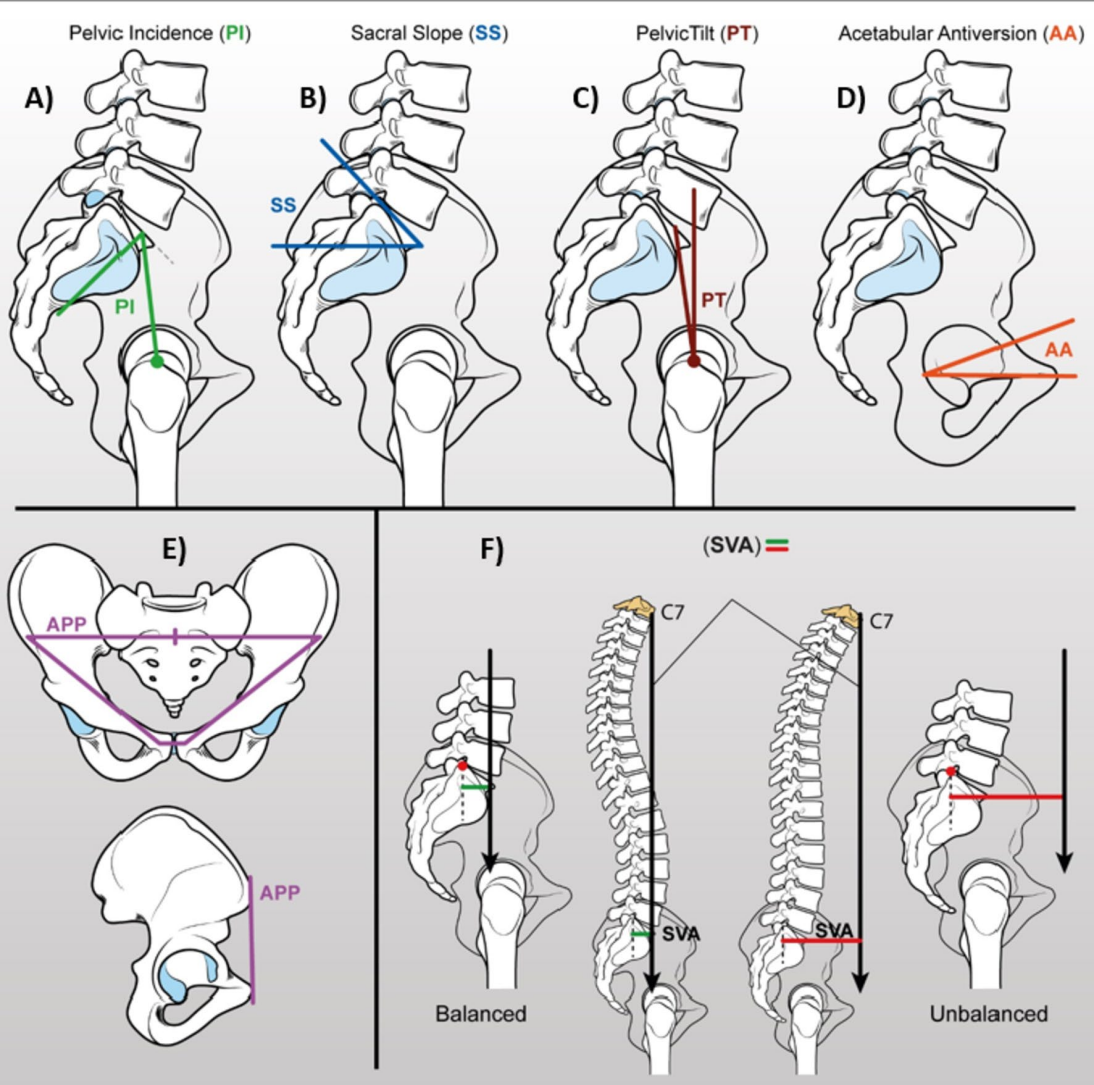
**A** Pelvic Incidence (PI): is an angle formed by a line perpendicular to the S1 endplate and a second line from the midpoint of S1 to the center of the bicoxofemoral axis. The PI value indicates the pelvis' ability to compensate the sagittal imbalance of the spine by rotation around the bicoxofemoral axis: the higher the PI, the greater the possibility of pelvic retroversion. It is equal to the sum of SS and PT; this relationship justifies the inverse relationship between SS and PT. **B** Sacral Slope (SS): is an angle formed by the S1 endplate and a horizontal line. High SS values imply a horizontal orientation of the sacrum (anteverted pelvis), while negative values make standing impossible. **C** Pelvic Tilt (PT): is an angle formed by a line from the midpoint of S1 endplate to the center of the distance between two femoral heads and a vertical line. It indicates the spatial orientation of the pelvis, which varies depending on the position assumed by the patient during standing and walking. It is complementary to SS: considering the rotation of the pelvis around the bicoxofemoral axis, PT increases when the pelvis rotates backward (retroversion) and decreases when the pelvis rotates forward (anteversion). **D** Acetbular Anteversion (AA): is an angle formed by a line through the long axis of the acetabulum and a horizontal line measured on sagittal radiographs. **E** Anterior Pelvic Plane (APP): a plane formed by the anterior–superior iliac spines (ASIS) and the pubic symphysis. In neutral spinal balance this plane corresponds to the Functional Pelvic Plane (FPP), a vertical plane through the pubic symphysis, the ASIS and perpendicular to the ground. **F** Sagittal Vertical Axis (SVA): Horizontal distance between a plumb line starting from the body of C7 and running perpendicular to the ground and the posterosuperior angle of S1. This parameter describes the overall sagittal balance of the thoracolumbar spine and it is considered physiological within 5 cm. Values between 5 and 15 cm outline a partial compensation, while decompensated alignment results above 15 cm

The evaluation requires the measurement of specific radiographic parameters of the entire spine and pelvis in sagittal view, namely Pelvic Incidence (PI), Sacral Slope (SS), Pelvic Tilt (PT), Acetabular Anteversion (AA), Sagittal Vertical Axis (SVA), Anterior Pelvic Plane (APP) (Fig. 2). Moreover, it may be useful to study the dynamics of the spinopelvic relationships through the variations of these parameters between the orthostatic and seated position.

There is a close relationship among the previously mentioned parameters [26, 27]. Le Huec and Hasegawa provided the cornerstone for the guidelines for sagittal spinopelvic balance defining the equations PI = PT + SS and PT = 0.44 PI−11° [27].

Moreover, to express the relationship between LL and PI, the most used equations are those of Schwab: PI = LL ± 11° [27] and Le Huec: LL = 0.54 × PI + 27.6° [26].

The quantification of spinopelvic motion, hip motion, acetabular positions, and femoral range of motion can be accomplished through the measurement of the abovementioned parameters on lateral radiographs of the lumbar spine and pelvis. This can be achieved by calculating the difference between two distinct postural positions, the standing and sitting positions. However, there are limitations to this method. First, despite pelvic twisting, the images must comply to the symmetry of anatomical reference points to avoid landmark overlap and a consequent difficult image evaluation [28]. Moreover, sagittal view may not be adequate to understand the functional evaluation of the “safe zone” [28]. Recent technologies that overcomes these limitations are the EOS X-rays equipment [29], and Computed tomography (CT) scans with 3D reconstructions. EOS™ biplanar low dose X-ray is a new imaging technique that provides 3D image of the skeleton in standing or sitting position, by simultaneously combining frontal and lateral acquisitions [30]. This technique reduces X-ray exposure and allows the evaluation of the overall postural abnormalities involving the spinopelvic area. CT scans are useful to understand the reciprocal morphology of acetabulum and proximal femur, but the exam/acquisition is usually performed in supine position and does not allow for functional evaluation of the patient [31].

For these reasons, specialized software were developed to create patient-specific 3D models. Adjusting the model according to the SS value on the lateral radiographs with the patient in standing and sitting position, allows for the simulation of the dynamic relationships between bones and implants [2, 32].

In patients with normal spinopelvic mobility, pelvis undergoes a posterior tilt while transitioning from a standing to a seated position. This posterior tilt facilitates a concomitant posterior tilting of the acetabulum, thereby creating space for the femur to flex towards the acetabulum. It is interesting to note that for each degree of posterior pelvic tilt (PT), there is a corresponding increase in the anteversion of the acetabulum by approximately 1 degree [33, 34]. This enables the femur to maximize flexion (towards the acetabulum) without any impingement [33–35].

Average range of motion involves the pelvis tilting posteriorly between 20° and 35° [36, 37] and the femur flexing around 55° to 70° [38, 39] with respect to the acetabulum. This motion results in an angle of roughly 90° between the femur and the upper body enabling an upright sitting posture. Nevertheless, as pelvic mobility declines, the femur must compensate by increasing its range of motion to accommodate postural adjustments [39–41]. A reduction in spinopelvic mobility results in a proportional increase in the range of femoral flexion required to achieve an upright sitting position. Specifically, for each degree of pelvic motion lost, an increase of approximately 1° in femoral flexion is necessary to enable the femur and upper body to attain the desired upright posture [42].

### Classifications of patients with HIP-SPINE deformity

Some descriptions for abnormal spinopelvic motion, spinopelvic deformity, and spinopelvic imbalance can be found in literature [39, 40].

The most accurate and thorough classification system to assess the relationship between spine and hips is the revised Bordeaux Classification of Spine-Hip Relations, developed by Rivière et al. [43]. To allocate patients into one of five categories (A, B, C, D, and F), this system correlates the Roussouly spine types [9], the acetabular types (defined by its anteversion), the spinopelvic parameters (calculated in a lateral full spine radiograph in both standing and sitting positions), and their mutual motion.

Category A represents a patient with healthy lumbopelvic complex and more than 10° of retroversion while seated. Category B indicates a stiffer lumbopelvic complex with less than 10° of retroversion when the patient is seated, which increases the chances of anterior impingement and posterior dislocation while sitting or squatting. In the last three categories (C–E), the patient has fixed pelvic retroversion when standing, stiff spine and sagittal balanced spine (C, compensated stage), or sagittal unbalanced spine (decompensation stage D). Finally, the patient could have a fused spine (F). This system allows the stratification of the risk of primary THA impingement or dislocation in three risk categories: A (very low to low risk), B–C (moderate to high risk), and D–F (very high risk) [45]. The complexity of the classification system and the lack of specific surgical indications for each category are its primary limitations, and therefore other authors developed simplified systems with a more direct impact on the surgical treatment.

Phan et al. [40] proposed a simple classification based on the PI–LL mismatch and the PT value as an index of spinal sagittal balance. They categorized four types of spines combining two principal characteristics: flexible or rigid, and balanced or unbalanced, including a straightforward treatment methodology with adequate indications for the most common hip-spine typologies of patients.

Other hip-spine classifications have been proposed to include every single subtype of hip-spine morphotype. Stefl et al. [18] identified 5 patterns of spinopelvic mobility based on the Sacral Slope: “Normal”, “Kyphosis” “Hypermobile Normal” with pelvic mobility of more than 30° between standing and sitting position, “Stuck Standing hips” with a pelvis fixed in anterior tilt, and “Stuck Sitting hips” with a pelvis fixed in posterior tilt. Hips that are stuck standing or stuck sitting are stiff: the changes of SS between standing and sitting position is ≤ 10° (∆SS < 10°); the “Fused Hips” are defined as a ∆SS ≤ 5° for biological or surgical fusion.

Luthringer and Vigdorchik [46] introduced a simplified Hip-Spine Classification for THA candidates. This system incorporates two important parameters of spinal deformity, PI-LL mismatch and SS. Patients are classified into two categories based on their spinal alignment: those with normal spinal alignment (PI-LL within ± 10°) and those with flatback deformity (PI-LL > 10°). Further classification is then performed based on spinal mobility, dividing patients into those with normal spinal mobility (∆SS > 10° between standing and sitting) and those with stiff spines (∆SS < 10°). Therefore, a total of four categories are formed by merging these characteristics: 1A, 1B, 2A, and 2B [46].

## Current evidence on “how to place implant”

The correct identification of hip-spine type patient allows the selection of the most suitable implants’ positioning for a specific patient. Patients are usually classified according to one of the systems outlined above. Classic parameters for cup placement in THA are approximately 40° cup of inclination and 20° of anteversion. Combined anteversion (calculated as the sum of version of cup and stem) is in the “safe zone” when values are within the 25–50° range [47, 48].

According to Phan classification [44], an algorithm for cup positioning is proposed according to the overall sagittal balance and spinopelvic parameters. In patients with normal sagittal alignment, we can find:

- 1A is Normal alignment with normal mobility: a pelvis with preserved spinopelvic mobility can be managed and treated with the traditional cup positioning of 20–25° of anteversion with an inclination of 40–45°.
- 1B is Normal alignment with stiff spine. Patients with normal sagittal alignment, where the anterior pelvic plane corresponds the functional plane, with a stiff spine (change of less than 10° in SS from stand to sit) need a more anteverted cup placement (approximately 30° of anteversion) to avoid the anterior impingement and consequent posterior dislocation. Moreover, in the coronal plane it is warranted a cup placement inclination of 45°.

Group 2 is defined as flatback deformity (with a PI-LL > 10°). Patients present a retroverted pelvis in standing position, and posterior pelvic tilt increases functional cup anteversion. Therefore, if the anatomical—instead of the functional—pelvic plane is considered, there is a risk to implant a too anteverted cup, increasing the risk of posterior impingement and anterior dislocation. In these patients, cup anteversion is targeted to the functional pelvic plane to allow cup placement within the safe zone:

- 2A flatback deformity (PI-LL > 10°) with normal mobility: anteversion should be targeted 25–30° form the functional pelvic plane (FPP) with a cup inclination of 40°.
- 2B flatback deformity (PI-LL > 10°) with stiff spine: more anteversion is required to prevent dislocation, so the advised anteversion should be with 30° of anteversion on the functional pelvic plane and a cup placement of 45°; these patients represent the population with higher risk of dislocation, and therefore the use of implants to decrease the risk of dislocation is advised [46, 49].

To include every hip-spine typology and the related cup placement, two more categories should be described. The first one being hypermobile hip-spine patients, presenting with a SS change above 30° from standing to sitting, in which greater acetabular coverage is required to reduce the verticality of the cup while sitting. In these cases a cup inclination of 35–40° and anteversion of 15–20° is advised to prevent dislocation [18]. The last category includes patients with increased anterior pelvic tilt and normal spinal mobility, typical of patients with hip flexion contracture. In these patients, it is required to follow the functional pelvic plane, with cup position targeted at 20–25° of anteversion with 40° of inclination [49].

## Three-dimensional models of the hip

Based on current evidence, it is possible to state that a standardized procedure does not ensure a correct placement of the implant. Patients differ from one another, and what works for one may not work for the others. Several factors (both anatomical and functional) may play a role in the success or failure of an implant. While planning the procedure, the expected range of motion of the implant and the expected post-operative level of mobility of the patient should be considered, as these parameters contribute to the success of the surgery. A mechanically sound and working implant that does not allow the patient to perform simple or common activities of daily routine will not be well perceived by the patient. However, it is not possible to test intraoperatively the actual range of motion of the implant.

For this reason, over the years, computer modelling and simulation techniques have been developed to support clinicians in the different steps of surgery [50]. For instance, 3D representations of the anatomical structures of interest, particularly bones and joints, may be employed to support and guide the pre-operative evaluation and to identify the best placement of the implant.

As medical images, typically CT data, are collected pre-operatively, anatomically accurate skeletal models can be generated. Such models, where the hip is typically represented as a ball-in-socket joint that allows for three rotations (i.e., intra/extra rotation, flexion/extension, and ab/adduction) but no translations, enable to quantify the residual (pre-operative) range of motion of the hip and to estimate the post-operative range of motion, taking into account the patient’s bony geometries, the shape of the implant, and its planned positioning. Computer simulations that employ skeletal models may be used to identify the joint configurations where bone-to-bone or bone-to-implant contact occur [51–53], therefore marking the contours of the so-called impingement-free zone. These models, also known as digital twins, further enable to test different scenarios, e.g. how the implant and the hip joint would move as the patient performs different activities of daily living (e.g. standing from a chair or walking). A digital twin can in fact be informed and/or guided by motion data collected in a gait laboratory (by means of motion capture systems) [54] or in the real world (via wearable sensors) [55], directly on the patient. Compensatory mechanisms may be highlighted, e.g. deteriorated spinopelvic alignment while walking in subjects with radiographic pelvic retroversion (compared to normal PT) [56]. Moreover, as several simulations may be performed in a limited amount of time, these methods may be employed to investigate the relationships between anatomical and functional (pelvic) parameters and the position and orientation of the implant components [57, 58]. Through a computational approach, Tang et al. [59] were able to test over 1600 hip joint configurations (i.e. movements), which enabled them to identify the impingement-free safe zone in 10 patients from CT data and EOS images (acquired in standing and sitting position). Undesired changes in spinopelvic parameters resulting from the surgical intervention could also be detected [15].

**Fig. 3:**
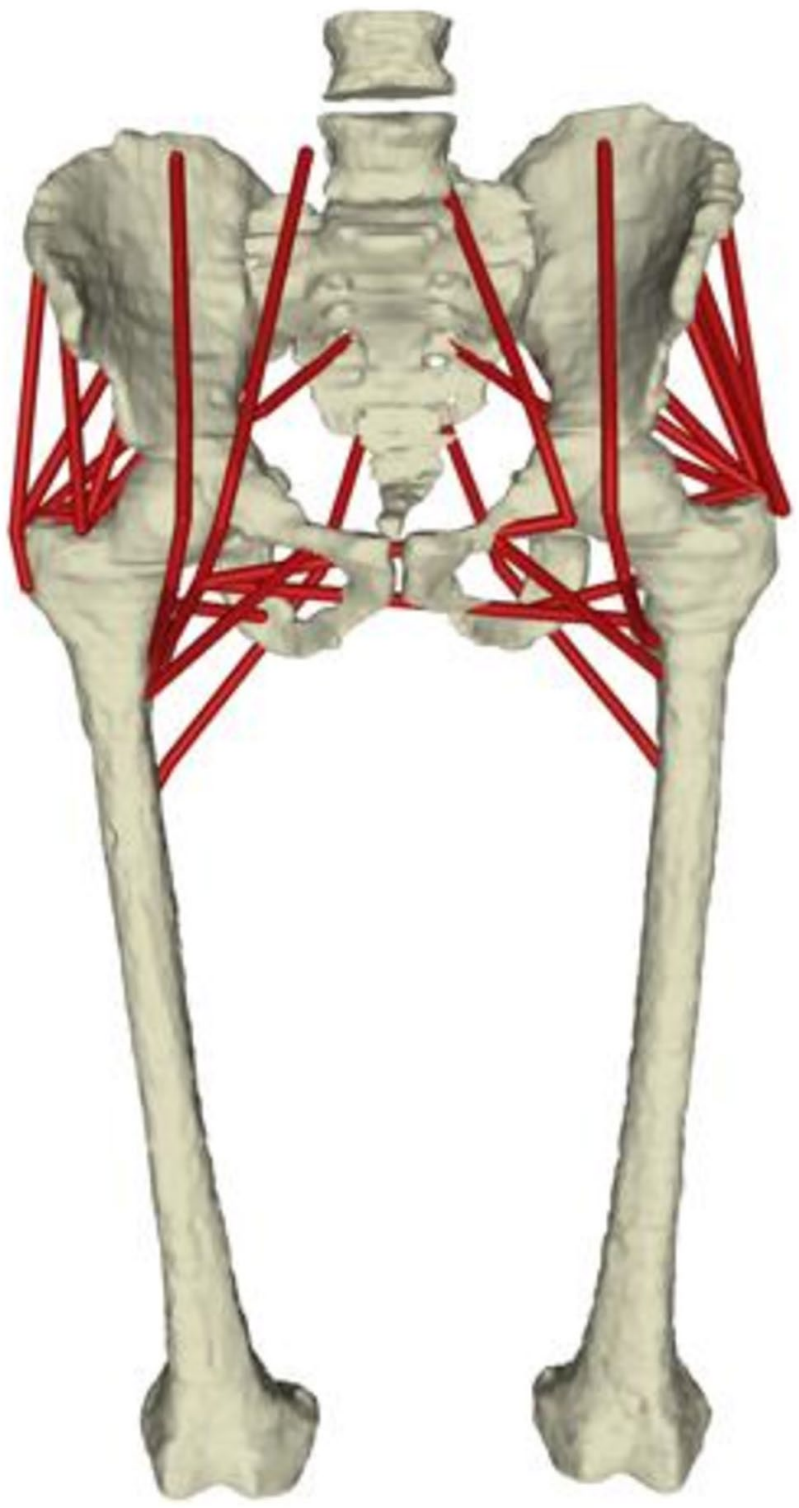
Example of image-based musculoskeletal model of the pelvis, hip and lumbosacral complex. Muscles are represented as red lines connecting origin and insertion points

When muscles were included into the models (Fig. 3), further analyses could be performed (e.g. to investigate spontaneous dislocations [60] or to estimate intersegmental loads at various joints). Personalised musculoskeletal models incorporating subject-specific spinopelvic parameters (e.g. PI, SS and PT) have been employed to explore the relationship between sagittal alignment and hip joint contact forces (i.e. forces transmitted at the hip level, between pelvis and femur bones) [61, 62] or lumbar loads [63]. Significant correlations between hip joint loads, SVA and femur obliquity angle were identified [62], but the same was not true for other parameters, including PI and SS. The SS, however, was found to affect compression forces and shear loads at lumbar level [63].

While promising, musculoskeletal models and computer simulations are not commonly used in clinical settings yet. This is due to the niche skillset required to develop the models and to run the simulations. To this end, simplified and streamlined workflows are under development to overcome the problem, and these are likely to become available in a relative near future [64].

### Finite element models

Different studies investigated the possibility to use Finite Element (FE) models to explore the hip—spine relationships. The computational models were typically obtained considering patient specific three-dimensional geometries of the bone segments [65–68] and simplified muscles and ligaments components [67, 68].

Applications ranged from biomechanical analyses of the spine-sacroiliac-hip complex to risk assessment studies of hip joint diseases due to spine pathological conditions after surgery. For example, Kitamura et al. explored how the change in sagittal pelvic tilt affects the loading environment and joint stress distribution in hip dysplasia [65]. In their study, 21 dysplastic hips and 21 normal hips were modelled using CT based patient-specific 3D FE models that included hemipelvis, the femur, and the acetabular and femoral cartilage components. The results obtained from these analyses suggested that the variation in physiologic PT may affect the mechanics within the hip joint, especially in dysplastic hips: while the average contact area decreased, the contact pressure and equivalent stresses increased as the pelvis tilted from 10° anterior to 10° posterior. Moreover, according to a finite element study published by Sakuma et al., the mechanical stresses at the normal hip joints increased as the posterior pelvic inclination increased. They demonstrated that the stress at the articular surface reached a level almost equivalent to that of hip joints with acetabular dysplasia at 25° [66]. In another study [67], a FE model of the spine-sacroiliac-hip complex was developed to investigate the effect of the sacroiliac joint fusion on the mechanical stress and contact area at the hip joint. The model was built considering boundary conditions corresponding to walking, rising, and descending stairs. Little changes in stress at the hip joint were observed after the segmental fusion, suggesting a low risk of developing the phenomenon of adjacent segment disease. More recently, Kumaran et al. used a spine-pelvis-hip FE model generated from CT images of a 55-year-old female patient to analyse the effects of changes in SS on the different biomechanical parameters, including hip joint stresses [68]. They simulated flexion, extension, lateral bending and axial rotation movements by varying the SS angle, and found that higher values of SS angle may increase stress on the hip joint.

## Discussion and treatment algorithm

The relationship between the spine and the hip has received significant attention from the scientific community, which has allowed the problem to be thoroughly examined over the last few years, highlighting critical aspects, and defining several clinical issues, many of which have found reasonable, and satisfactory solutions. However, there remains a certain difficulty in approaching this problem, especially for those unfamiliar with issues related to spinopelvic relationship and THA instability or dislocation. Additionally, there is an evident lack of standardization in terms, objectives, and therapeutic actions, making it challenging to address the problem systematically. Clinical recommendations often provide little practical guidance, failing to offer a simple and clear approach to the problem [69, 70].

The following general recommendations may improve outcomes and ease patients’ evaluation and management also by less experienced surgeons.

### Aim to increase anteversion

Most of current working classification systems describe targets for cup anteversion during THA surgery. However, except for navigated or robotic THA performance, surgeons are not able to precisely correct 5° of anteversion during cup placement [71]. Moreover, in most patients, an increase in cup anteversion with respect to the functional pelvic plane is required to decrease the risk of dislocation, reaching 25–30° of cup anteversion in most patients [48]. Not all surgical approaches are equal in accomplishing an increased intraoperative cup anteversion. Surgical approaches requiring supine patient positioning, including anterior, anterolateral and direct lateral approaches are associated to more anteverted cup positioning compared to posterolateral approach [72].

**Fig. 4:**
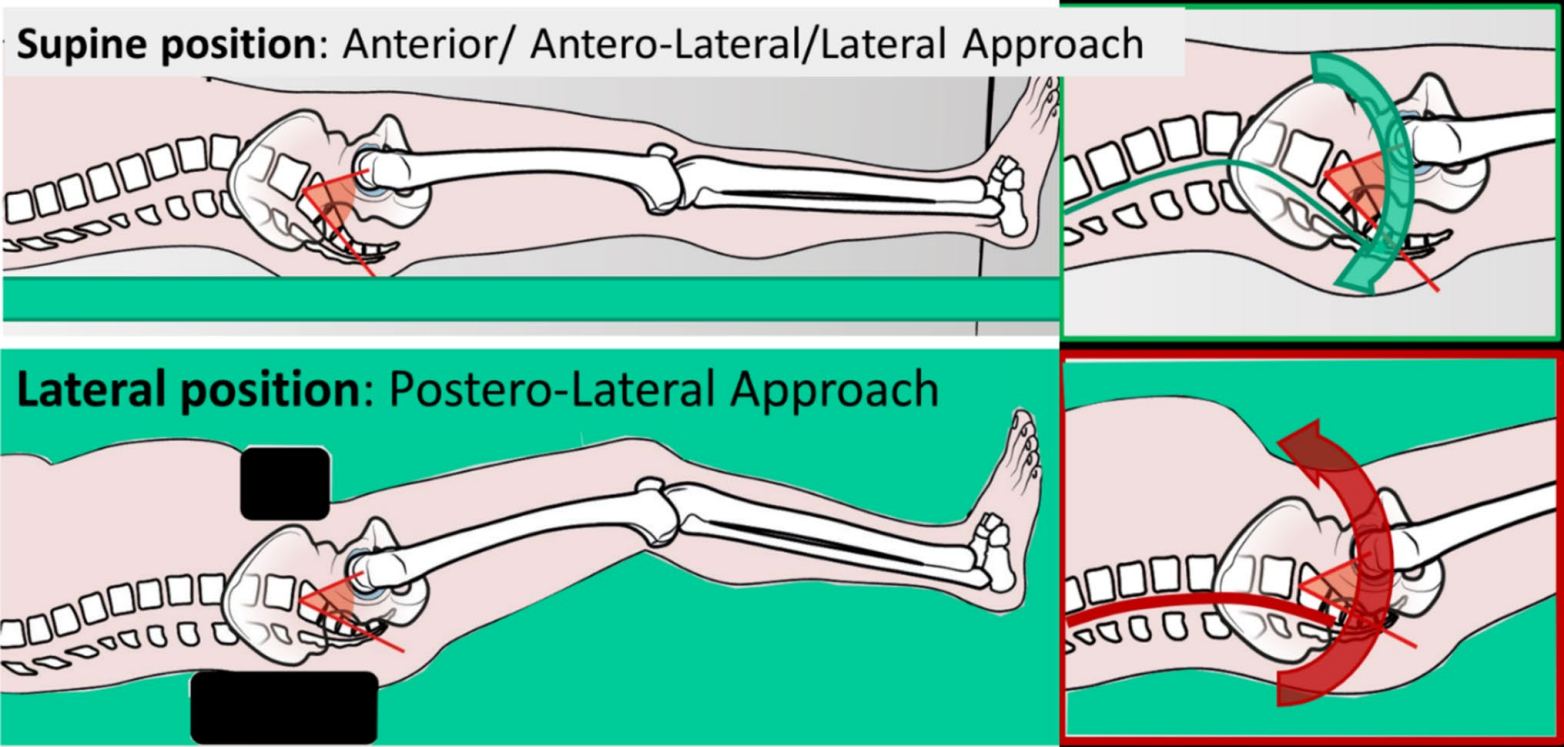
the pelvis position changes in relation to the surgical placement, in the supine position, the natural pelvic alignment is maintained, while in the lateral decubitus, the pressors place the pelvis in a more retroverted position

This is mainly due to patient positioning on the surgical table (Fig. 4); in fact, in patients laying down in lateral decubitus during THA surgery, as in posterolateral approach, pressors are posed on the sacrum and thighs are flexed, determining an increase in pelvic retroversion and a tendency to less anteverted cup positioning with eccentric reaming of the posterior acetabulum, being responsible for the increased rate of posterior dislocations with this surgical approach. Among the supine based surgical approaches, anterior-based approaches (direct anterior and anterolateral) are usually associated to more anteverted cup positioning respect with direct lateral approach [73].

### Balance is more important than stiffness

Orthopaedic surgery is a surgical discipline, and worldwide more and more surgeons are skilled in surgery of one body site, leading to a decrease in the overall evaluation of the patients for diseases at adjacent districts. In this scenario, the knowledge of the hip-spine relationship and evaluation is now a crucial requirement for both hip and spine surgeons. Functional patient evaluation during ambulation allows the clinician to understand pelvic position in space and functional pelvis plane orientation while standing. Unbalanced patients have more retroverted pelvis and require more anteverted cup placement [48, 49, 69].

**Fig. 5:**
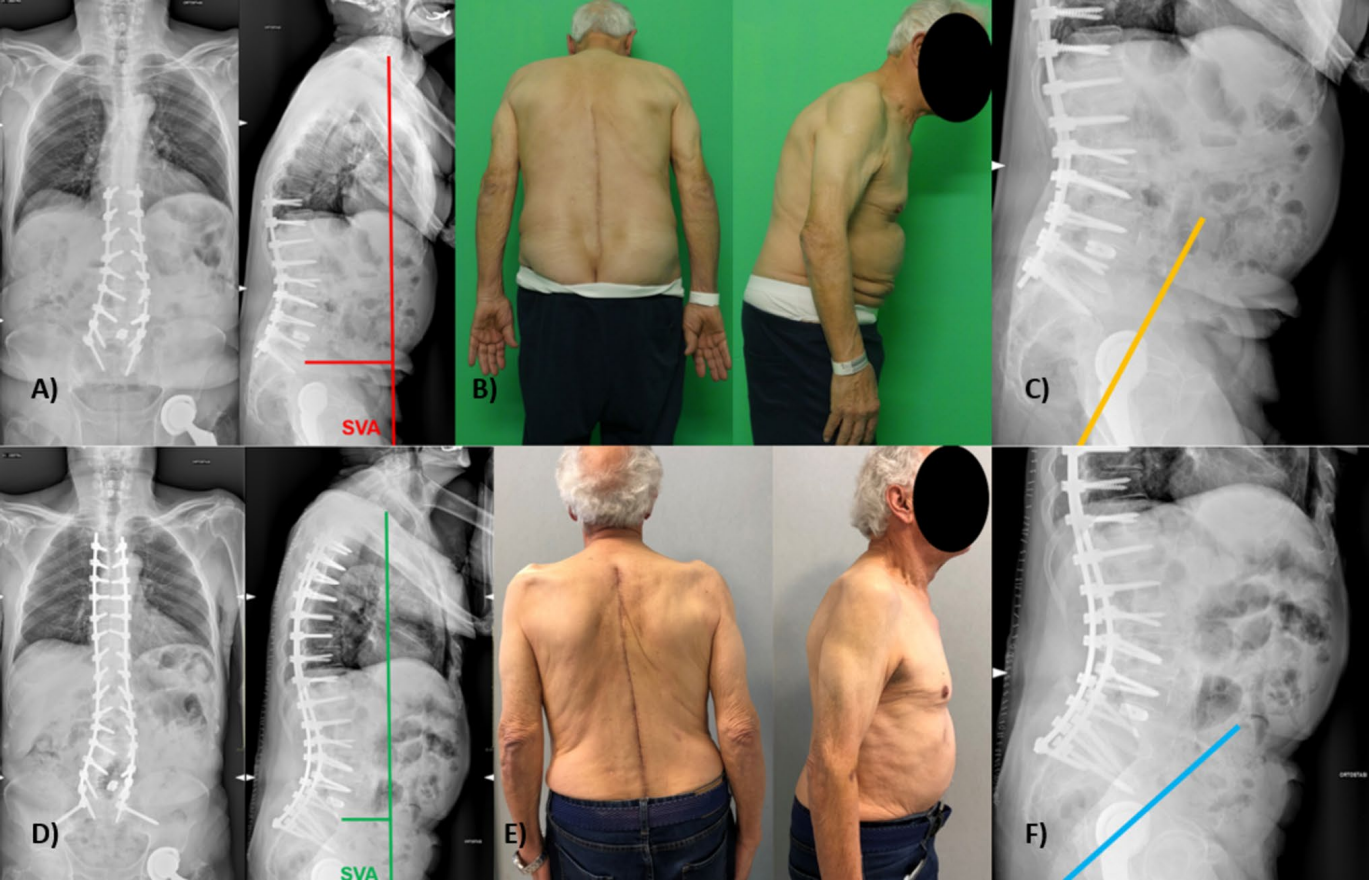
A 72-years old patient, with a previous spine surgery and a THA, with a sagittal unbalancing, measured as 16 cm in SVA (**A**, **B**) and a consequent augmented AA with a value of 66° (**C**). A spine revision surgery was performed to extend proximally the arthrodesis area, correcting the sagittal balancing, improving SVA with a value of 7 cm (**D**, **E**) and the improvement of spinopelvic parameter with an AA of 38° (**F**)

Patients with LSF are more frequently associated to mechanical complications after THA. In many patients, if lumbar lordosis is not adequately restored after LSF surgery, there is an increased risk of adjacent segment degeneration of the spine [22], and a worsening of overall alignment with pelvis retroversion and increased risk of posterior impingement and risk of dislocation of THA implants [74]. This can be corrected at least partially when revision lumbar spine surgery is performed, with improvement of spinopelvic parameters and restitution of overall balance (Fig. 5).

**Fig. 6:**
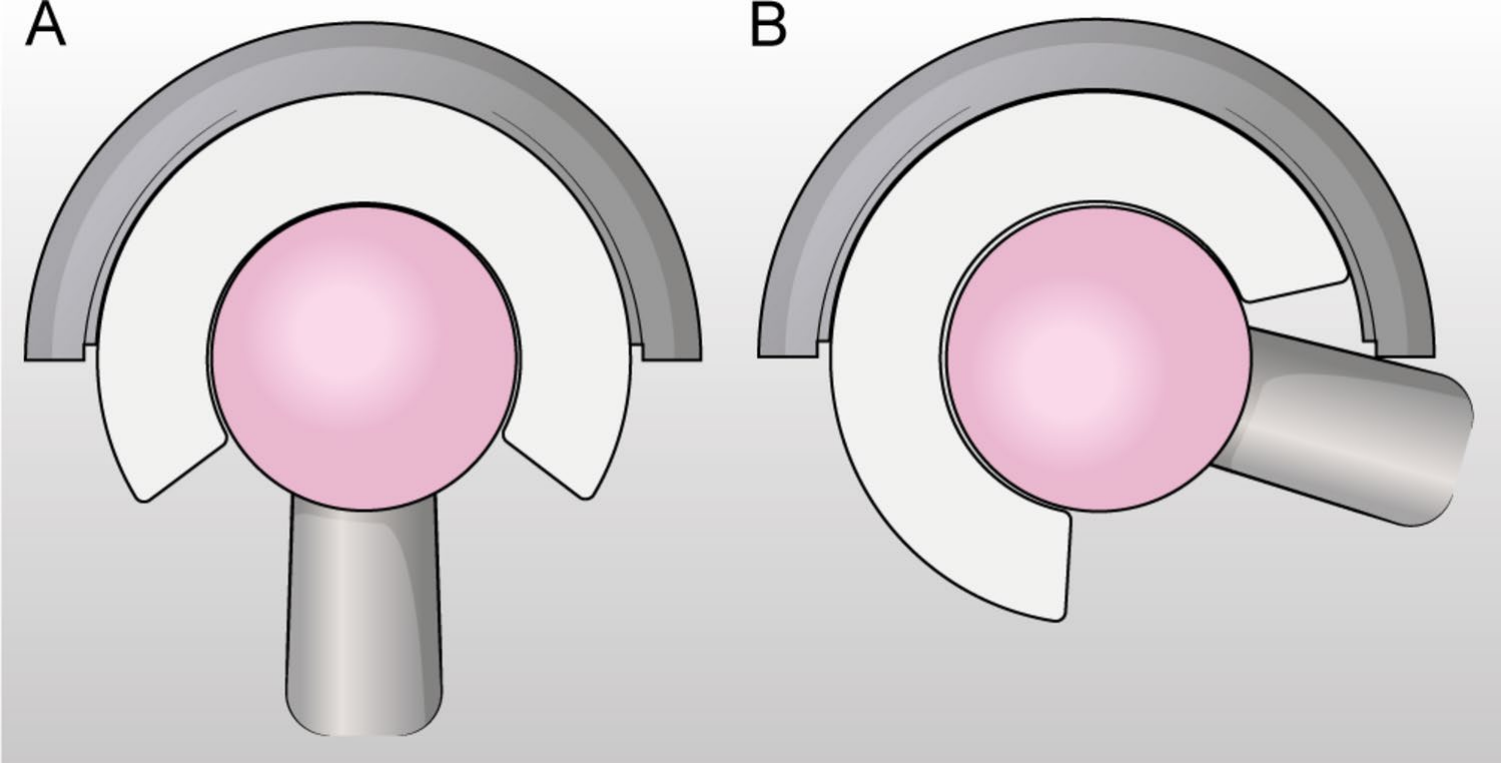
Dual Mobility implant has two points of articulation: one between the shell and the polyethylene (external bearing) and one between the polyethylene and the femoral head (internal bearing). The inner bearing moves; the outer bearing moves only at extremes of movement

If spinal surgery is not planned and the patient is unbalanced, the use of a dual mobility implant may be a solution to decrease the risk of dislocation [69, 75, 76]. Dual mobility implants work by increasing the jumping distance and, at the same time increase the range of motion of the joint, thereby reducing the risk of impingement and subsequent dislocation; this is achieved because in the double articulation the liner works as a bigger femoral head, protecting the risk of impingement because of the greater range of motion allowed by the sum of the two joints [75, 77, 78] (Fig. 6).

In those few patients with stuck sitting or stuck standing deformity, the use of increased offset necks can decrease the risk of impingement, and may be a useful tool to decrease wear by local conflict and the overall risk of failure [79].

**Fig. 7:**
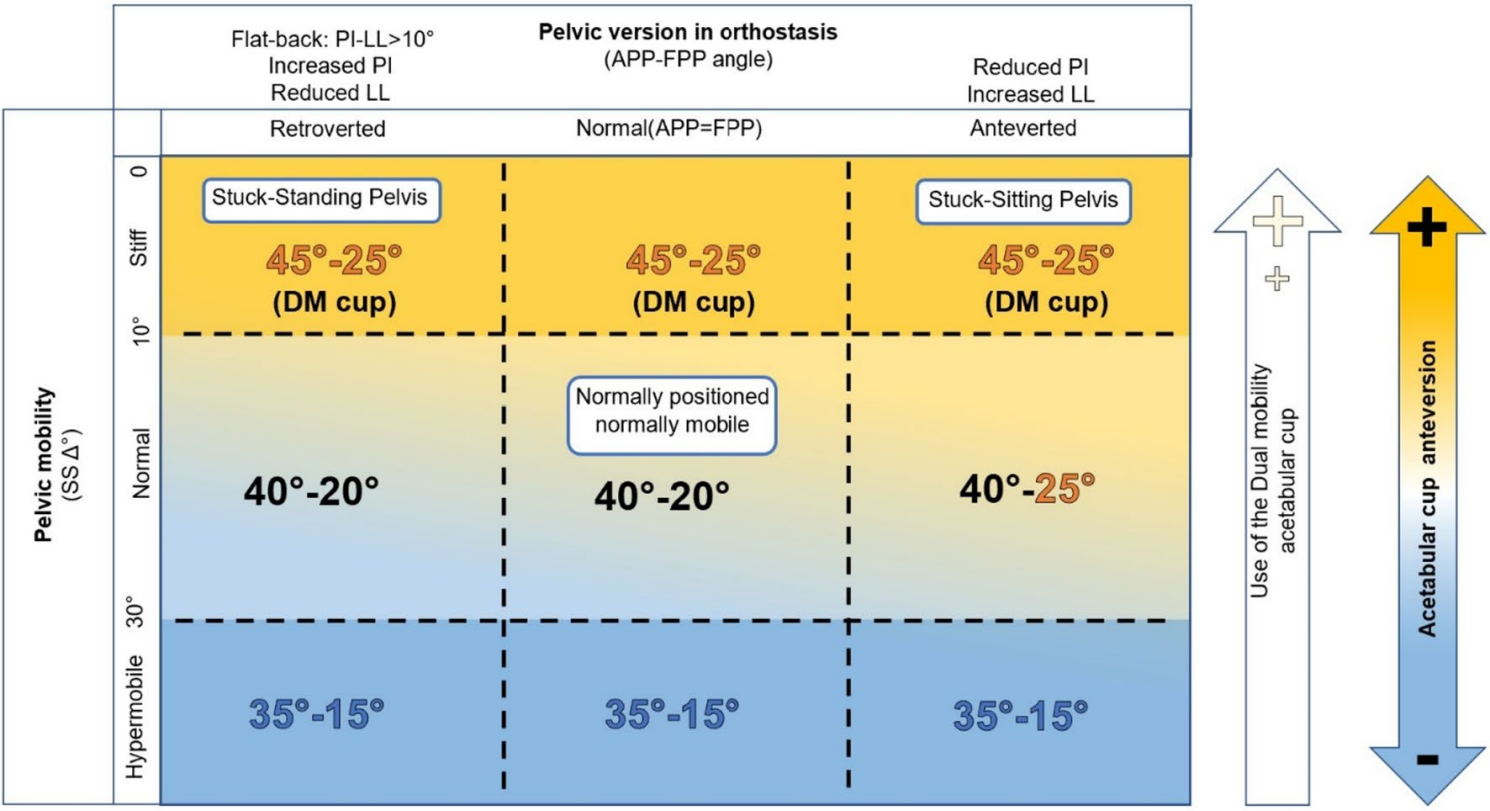
Recommendations for total hip arthroplasty performance: in each quadrant it is indicated the recommended position for acetabular cup, namely inclination and anteversion, considering the APP

Summarizing all these considerations we developed a graphical algorithm to guide surgeons in decision making in THA in patients with spinopelvic issues (Fig. 7).

The recommendations were compiled and presented in a chart that delineates 9 different patterns based on pelvic version and mobility parameters. For each parameter, three types of lumbo-pelvic complexes were identified. Pelvic version parameters distinguish among normally aligned, retroverted, and anteverted pelvis in a standing position. The pelvic mobility parameter classifies the pelvis as normo-mobile, hypermobile, or stiff (with limited mobility up to the fixed pelvis). A stiff pelvis requires greater anteversion and inclination, along with the use of dual mobility cups. Normo-mobile anteverted pelvis requires increased anteversion, while in the hypermobile pelvis less anteversion and inclination is warranted, even though hypermobile pelvis patients usually find a balance and patients are rarely symptomatic. Stuck-standing and stuck-sitting patients, in which recommendations about the positioning of acetabular component are not sufficient to guarantee implant stability and reduce the risk of impingement and dislocation, may benefit from the use of lateralized (increased offset) femoral components. These recommendations take into account the multiparametric nature of pathological changes in hip-spine relationship, and it must be associated with additional strategies aimed at ensuring a stable artificial joint; these parameters might be the base for machine learning and artificial intelligence software to guide surgeons and robotic hip surgery in the future.

## Conclusion

An increase in patients vulnerable to mechanical complications of THA implants because of pathologic hip-spine relationship, as LSF patients, is expected in the future. Therefore, despite recent technological advancements in both the diagnostic and therapeutic settings, this issue will continue to be present, and significantly impact clinical practice. For this reason, an effort is required to give surgeons an easy tool to address patients hips and spines, and to ease the choice of surgical approach and THA implants.
